# Cord blood red cell concentrates for preterm neonate transfusion: Insights from the multicenter BORN trial

**DOI:** 10.1111/trf.70241

**Published:** 2026-04-29

**Authors:** Claudio Pellegrino, Patrizia Papacci, Carlo Dani, Francesco Cresi, Giulia Remaschi, Giulia Ansaldi, Carmen Giannantonio, Maria Francesca Campagnoli, Barbara Vania, Marco Fabbri, Roberta Penta de Vera d' Aragona, Anna Molisso, Enrico Beccastrini, Antonella Dragonetti, Tina Pasciuto, Sabrina Gabbriellini, Silvia Baroni, Francesca Serrao, Velia Purcaro, Genny Raffaeli, Stefania Villa, Daniele Prati, Isabella Mondello, Alessandra Falcone, Maria Letizia Patti, Tiziana Boggini, Paola Bergamaschi, Iolanda Mozzetta, Caterina Giovanna Valentini, Emanuela Locatelli, Roberto Albiani, Federico Genzano Besso, Giulia Vanina Cantone, Alessandra Coscia, Alfonso Trimarchi, Anita Capone, Stefano Ghirardello, Giovanni Vento, Luciana Teofili

**Affiliations:** ^1^ Fondazione Policlinico A. Gemelli IRCCS Rome Italy; ^2^ Università Cattolica del Sacro Cuore Rome Italy; ^3^ Azienda Ospedaliero Universitaria Careggi Florence Italy; ^4^ Città della Salute e della Scienza Torino Italy; ^5^ Department of Public Health and Pediatrics Università di Torino Torino Italy; ^6^ Azienda Ospedaliero Universitaria Pisana Pisa Italy; ^7^ Ospedale Santobono Pausilipon Naples Italy; ^8^ Ospedale Evangelico Villa Betania Naples Italy; ^9^ Fondazione IRCCS Ca' Granda Ospedale Maggiore Policlinico Milan Italy; ^10^ Department of Clinical Sciences and Community Health Università di Milano Milan Italy; ^11^ Grande Ospedale Metropolitano Bianchi Melacrino Morelli Reggio Calabria Italy; ^12^ Fondazione IRCCS Policlinico S. Matteo Pavia Italy; ^13^ Presidio Ospedaliero di Pescara Pescara Italy

**Keywords:** extremely low gestational age neonates, fetal hemoglobin, randomized controlled trial, retinopathy of prematurity, transfusions, umbilical blood

## Abstract

**Background:**

The BORN trial suggested that using cord blood‐red blood cells (CB‐RBCs) to transfuse severely preterm neonates significantly improves clinical outcomes compared to standard adult donor RBCs (A‐RBC).

**Study Design and Methods:**

The study illustrates CB‐RBC concentrate production and inventory management across nine CB banks participating in the BORN trial. Quality requirements were those established by the European regulation for leukocyte‐depleted RBC concentrates in additive solution. The compliance rate among centers was reported.

**Results:**

During the BORN study, 451 CB‐RBC units were processed and 107 were transfused to 69 patients in the intervention arm. However, for 67 transfusion requests, no compatible CB‐RBC units were available and adult‐RBCs were given. The CB‐RBC inventory comprised a minor fraction of CB units collected during the study period, suggesting that many did not meet the protocol‐defined volume threshold of 67 mL at collection. Quality control results showed that 84.0% of units achieved target hematocrit (Htc) levels (50–70%), with higher success rates in centers processing more CB units. All centers met quality standards, maintaining residual leukocytes below 1 × 10^6^ and end‐storage hemolysis below 0.8% in more than 90% of cases. CB‐RBC availability was significantly limited by the time required for bacterial testing results and the need for γ‐irradiation.

**Discussion:**

These data suggest that the standardized CB‐RBC production is reproducible. Moreover, extending CB‐RBC storage to 21 days could maximize the inventory utility.

**Abbreviations**
A‐RBCs: adult donor red blood cells
BPD: bronchopulmunary dysplasia
CB: cord blood
CB‐RBCs: cord blood Red blood cells
ELGANs: extremely low gestational age neonates
HbA: adult hemoglobin
HbF: fetal hemoglobin
RBCs: red blood cells
ROP: retinopathy of prematurity

## 1 INTRODUCTION

Clinical research in neonatal transfusion has recently challenged the preexisting dogma guiding blood product administration. Particularly in the clinical setting of prematurity, the lack of evidence of the beneficial impact of the liberal use of blood products led to reduced transfusion thresholds for both red blood cell (RBC) and platelet concentrates, while a few indications are nowadays recognized for administering plasma.^1^, ^2^, ^3^, ^4^ Likewise, a growing body of evidence established a connection between transfusions and poorer outcomes. Among the underlying pathogenetic mechanisms, the oxidative stress conveyed by blood products themselves seems to be implicated.^5^ One of the consequences of RBC transfusion in extremely low gestational age neonates (ELGANs) is the non‐physiological replacement of fetal hemoglobin (HbF) by the adult one (HbA).^6^ Repeated RBC transfusions abnormally increase the HbA levels, and due to the lower oxygen affinity of HbA, it results at the cellular level in a hyperoxic challenge.^7^ This process likely worsens the clinical course of the so‐called “oxidative stress‐related diseases,” a group of conditions typically observed in these fragile patients endowed with an impaired antioxidant response.^8^

In a study carried out in 2015, we demonstrated the feasibility of transfusing preterm neonates with RBC concentrates obtained from an allogeneic solidary donated cord blood (CB) unit.^9^ Subsequently, in the proof‐of‐concept CB‐Trip trial, we showed that CB‐RBC concentrates raise the Hb concentration without affecting physiological HbF levels.^10^ Finally, in the subsequent BORN trial, we have found that transfusing ELGANs with CB‐RBCs in their early postnatal life reduces the incidence of more severe forms of retinopathy of prematurity (ROP)^11^ and bronchopulmonary dysplasia (BPD),^12^ the most frequent oxidative stress‐related diseases that can adversely affect the long‐term quality of life of these patients.^13^

During the abovementioned studies, we refined the methods to process whole CB units into RBC concentrates, to establish the minimum volume of processable units and maximize RBC mass recovery even from low‐volume units. Moreover, the BORN trial posed us the challenge of getting reproducible standards among the different CB banks distributed across the national territory. Considering that only one CB bank operates per region, comparable multicenter operational data are essential to support the clinical translation of this transfusion strategy. This manuscript focuses on the manufacturing feasibility and operational implementation of a CB‐RBC inventory for patients in the BORN trial. The information provided can represent a model for future studies on CB‐RBC transfusions as well as the basis for implementing this groundbreaking transfusion approach into clinical practice.

## 2 MATERIALS AND METHODS

### 2.1 Study design

Study design and outcomes have been previously reported.^13^, ^14^ Briefly, BORN was a prospective, randomized, multi‐center, double‐blinded, controlled trial enrolling preterm neonates delivered at a gestational age (GA) between 24 + 0 and 27 + 6 weeks. Patients were recruited at eight Neonatal Intensive Care Units (NICUs), and CB units were provided by nine CB banks belonging to the Italian Cord Blood Bank Network. The study was approved by the Ethics Committee of Fondazione Policlinico A. Gemelli IRCCS (ID 4364, Prot. N. 003590/21) and of all participating centers and was registered at https://clinicaltrials.gov (NCT05100212). Patients were randomized 1:1 to receive until 29 + 6 weeks postmenstrual age (PMA) either adult donor‐RBCs (A‐RBCs, control arm) or allogenic CB‐RBCs (intervention arm). If compatible CB‐RBC units were not available, A‐RBC units were administered. After 29 + 6 weeks of PMA, all patients received A‐RBCs. Both A‐RBC and CB‐RBC units were matched for ABO/RhD blood group antigens and γ‐irradiated at distribution. Randomization was performed at blood banks through the Research Electronic Data Capture (REDCap) (RRID:SCR_003445) tool hosted at Fondazione Policlinico Universitario A. Gemelli, IRCCS (https://redcap-irccs.policlinicogemelli.it/). The arm allocation was not visible to neonatologists. To maintain blinding, A‐RBCs and CB‐RBCs were distributed in the same type of bags. The primary outcome was the rate of severe ROP (defined as ROP stage 3 or higher in zone I or II and/or the presence of plus disease) at discharge or at 40 weeks of PMA, whichever occurred first.

### 2.2 CB‐RBC collection and processing

**FIGURE 1:**
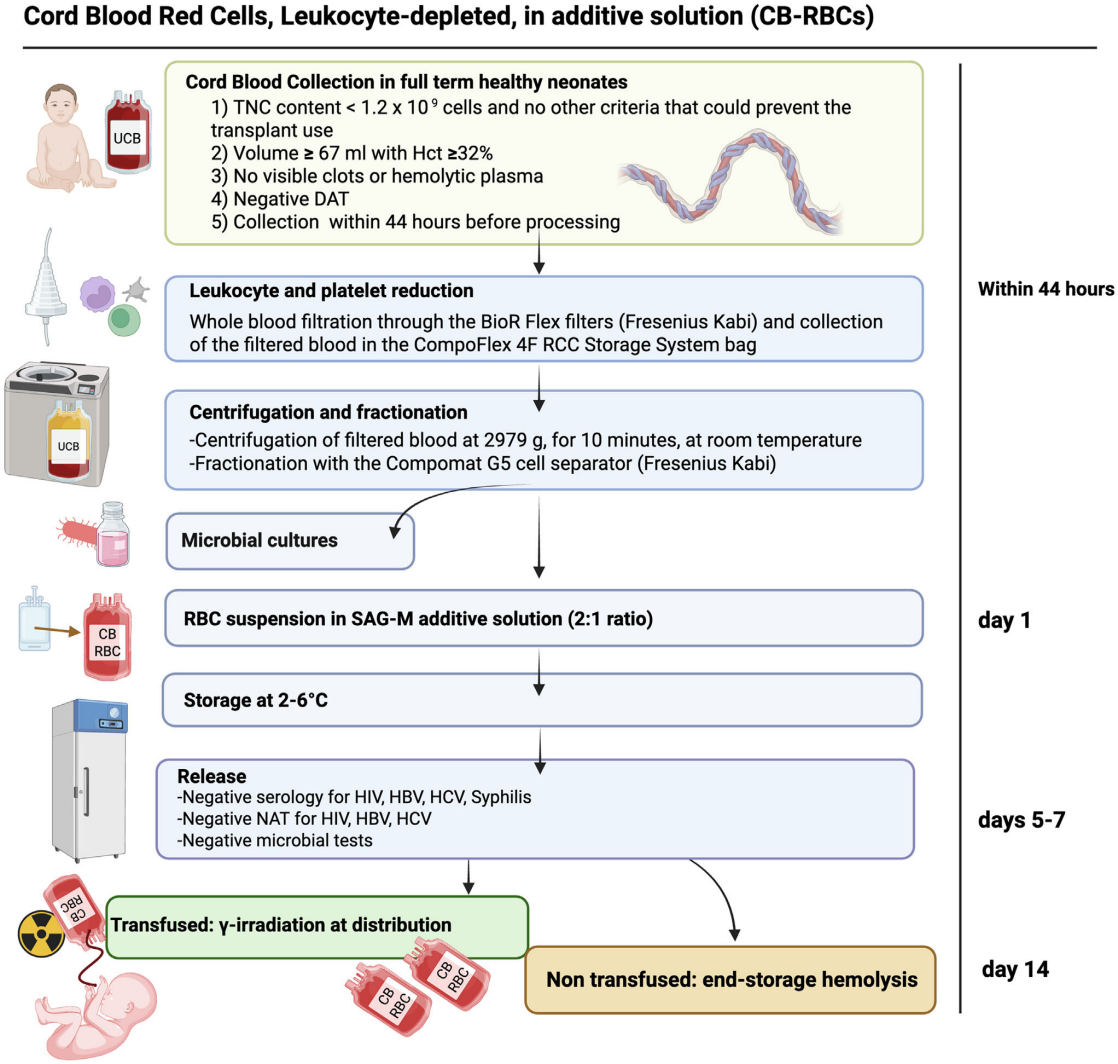
Main eligibility parameters adopted in the BORN study for processing CB units into RBC concentrates. Processing was performed within 44 h of collection. After leukodepletion and centrifugation, aliquots of RBCs and plasma were used for bacterial and fungal culture tests. RBCs were suspended in SAG‐M additive solution and stored at 2–6°C (day 1). CB‐RBC units were released after microbial tests were negative (day 5–7), and units were transfused by day 14 to allow γ‐irradiation. The units not used for transfusion were used to assess the end‐storage hemolysis rate. CB, cord blood; SAG‐M, saline, adenine, glucose, mannitol; RBC, red blood cells.

Figure 1 summarizes the flow of CB units from collection to transfusion or discard. Donation eligibility was defined according to the Italian regulation on CB hematopoietic stem cell donation. During pre‐donation counseling, couples were informed that CB units deemed unsuitable for transplantation could be processed into RBC concentrates and used to transfuse preterm neonates enrolled in the BORN study. A minority of CB banks adopted a specific consent form, whereas most local Ethics Committees deemed the national informed consent adequate, as it encompassed the research use of cord blood units not suitable for transplantation. CB units were collected in 30 mL of CPD. Criteria for processing into RBC concentrates were (1) total nucleated cell content <1.2 × 10^9^ cells and no other criteria that could prevent the transplant use according to Italian regulation (i.e., maternal or paternal history of cancer, autoimmune hereditary or genetic diseases, infectious diseases during pregnancy)^15^; (2) volume >67 mL with Hct >32%; (3) no clots or hemolytic plasma; (4) negative direct antiglobulin test; and (5) collection performed within 44 h before processing. The staff responsible for the processing were properly trained before the study. All units were kept stored at 2–6°C until processing, which encompassed (a) the whole blood filtration through the BioR Flex filters (Fresenius Kabi) and collection of the filtered blood in the CompoFlex 4F RCC Storage System bag; (b) the centrifugation of filtered blood at 2979*g*, for 10 min, at room temperature; (c) the fractionation with the Compomat G5 cell separator (Fresenius Kabi) with two different prespecified programs depending on whether the post‐filtration volume was < or >80 mL; (d) the RBCs recovery and suspension in saline, adenine, glucose, mannitol (SAG‐M) additive solution in a 2:1 ratio. The quality standards for CB‐RBCs were the same established by the European regulation for leukocyte‐depleted RBC concentrates in additive solution and included residual leukocyte content after filtration <10^6^ cells (as evaluated by flow cytometry according to center standard procedures), Hct 50–70%, and end‐storage hemolysis rate <0.8% of the initial Hb content in a minimum of 90% of units tested.^16^ After processing, all CB‐RBC concentrates were tested for bacterial and fungal contamination, using pediatric BacT/Alert blood culture bottles and automated detection system (Biomèrieux, Marcy‐l'Etoile, Lyon, France), and stored at 2–6°C temperature. Criteria for release were: negative serology for human immunodeficiency virus (HIV), hepatitis B virus (HBV), hepatitis C virus (HCV), and syphilis, and negative nucleic acid test for HIV, HBV, and HCV on maternal plasma samples collected at the time of delivery, and negative microbiological test. The maximum storage length was 14 days to allow γ‐irradiation.^16^ Non‐transfused units were used to assess the end‐storage hemolysis level (Figure 1).

### 2.3 Data collection

Data were collected in REDCap in a dedicated eCRF. Parameters at collection (date and time, volume, complete cell blood count [CBC], ABO/Rh typing), at processing (date and time, RBC concentrate volume, CBC, residual leukocytes, free hemoglobin), and at the end of the storage (CBC, free hemoglobin) were recorded.

### 2.4 Statistical analysis

Continuous variables were expressed as median and relative 25th‐75th percentile range (interquartile range [IQR]), and categoric variables as proportions (*n*, %). For differences between groups Fisher's exact test for categorical variables and the Mann–Whitney *U* test for continuous variables were use. Correlation was expressed by the Spearman's or Pearson's correlation coefficient, as appropriate (*r*). Two‐sided tests were applied, and the statistical level of significance was set at 0.05. Quality control measurements were performed on subsets of units. All available quality control data were included without imputation of missing data and results were reported based on the number of units tested. NCSS10 Statistical Software 2015 (NCSS, LLC., Kaysville, Utah, USA, ncss.com/software/ncss) and GraphPad Prism version 10.0.0 (GraphPad Software, Boston, Massachusetts, USA, www.graphpad.com) were used.

## 3 RESULTS

### 3.1 CB‐RBC inventory and the impact on study management

**FIGURE 2:**
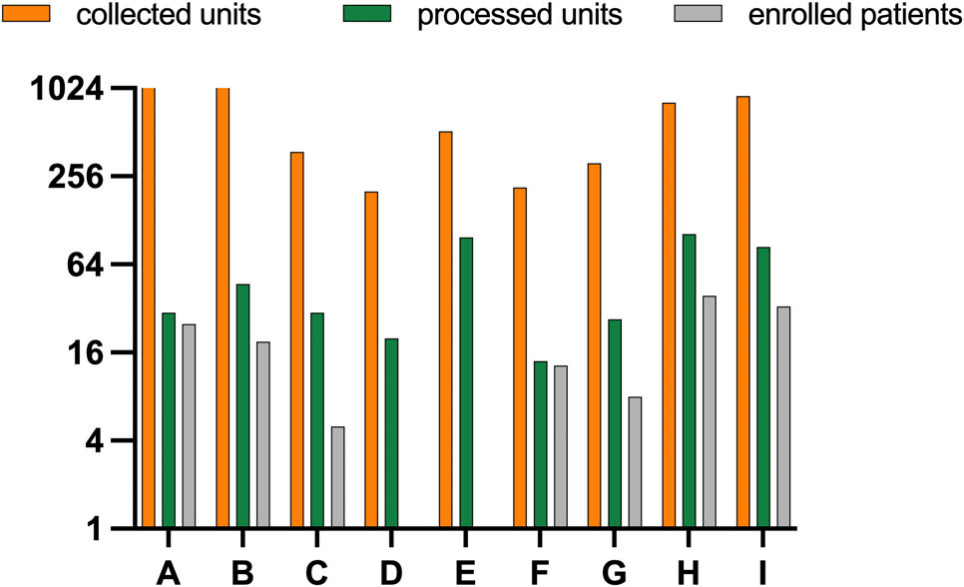
Total number of cord blood units collected at participating banks, number of units processed into CB‐RBC concentrates for the BORN trial, and number of enrolled patients in the corresponding NICUs, when present. Data are given on a base‐2 logarithmic scale. CB‐RBCs, cord blood‐red blood cells; NICUs, Neonatal Intensive Care Units.

**FIGURE 3:**
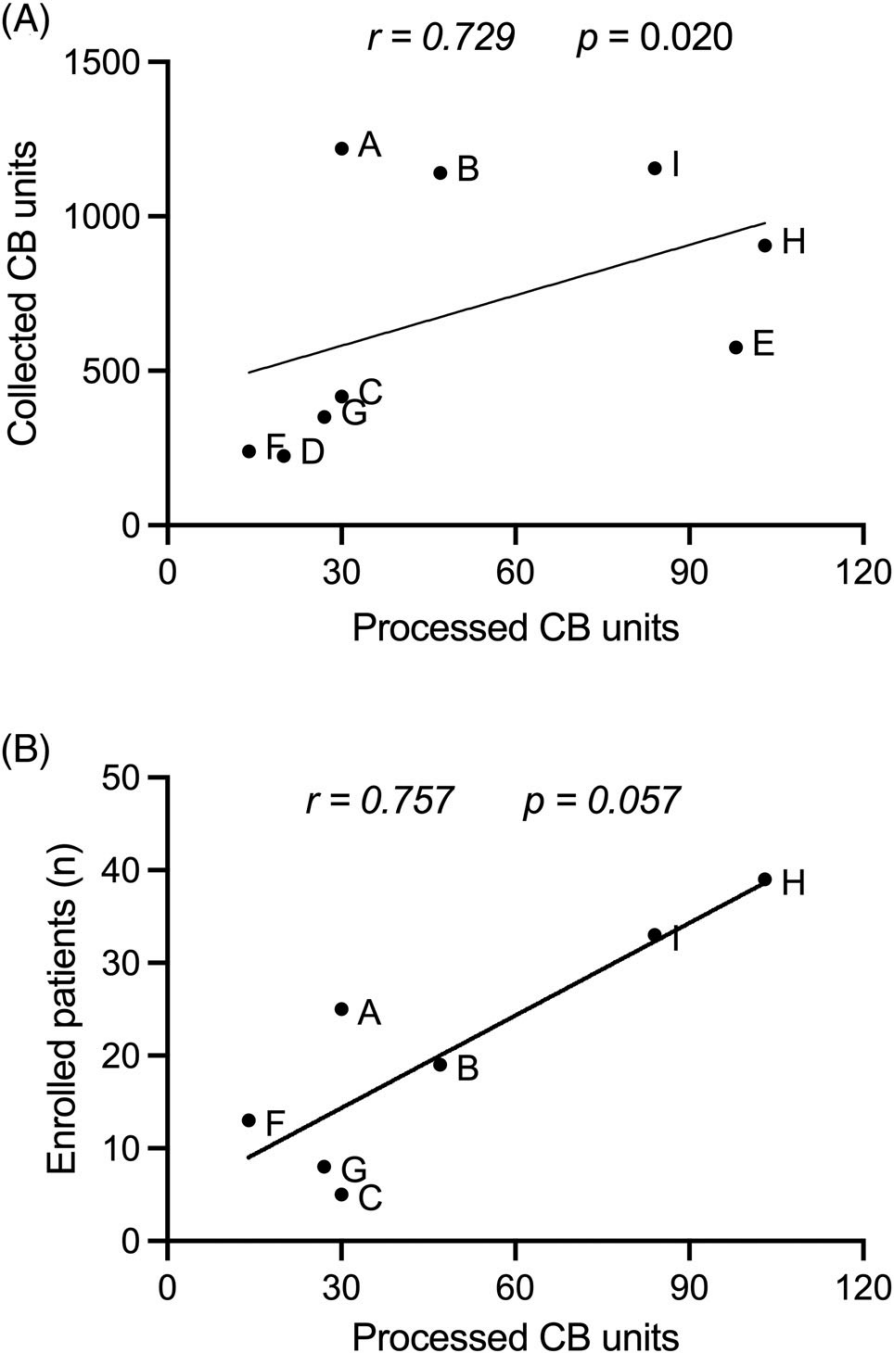
Number of cord blood units processed at different centers. (A) Correlation with the number of units collected at cord blood banks. (B) Correlation with the number of patients enrolled at the corresponding neonatal intensive care unit.

The BORN study started in December 2021 and ended in December 2024. During the 3‐year study period, 451 CB‐RBC units were produced by nine CB banks. A total of 18 units (3.9%) were found positive at microbial testing and were therefore not released: the positivity rate among centers ranged from 0% to 10.3% (*p* < 0.001 at χ^2^ test). The number of units processed was only a minor fraction of that of 5467 units collected at the same sites during the study period (data of the Italian Cord Blood Network available at https://www.centronazionalesangue.it/rapporti-di-attivita-delle-banche-sco/) (Figure 2): the average proportion of collected CB units processed into CB‐RBCs was 8.4% (IQR 6.0–10.6), with a high variability among centers, ranging from 2.7% to 18.9% (*p* < 0.001 at χ^2^ test). This finding could be explained only in part by the decision to prioritize O‐group units for fractionation (70.5%, Table 1). The number of processed units was proportional to the amount of CB units collected at CB bank (*r* = 0.742, Figure 3A) and of patients enrolled at the corresponding NICU (*r* = 0.757, Figure 3B).

**TABLE 1:** Parameters of the 451 cord blood units collected and processed in the 9 blood banks participating in the BORN study.

	A	B	C	D	E	F	G	I	H	TOT
	*N* 30	*N* 47	*N* 30	*N* 20	*N* 98	*N* 14	*N* 25	*N* 84	*N* 103	*N* 451
Volume, mL	89.0 (81.7–99.3)	103.0 (95.0–118.0)	88.5 (69.5–115.5)	94.0 (83.8–102.8)	83.5 (73.0–99.2)	69.0 (58.7–88.0)	88.0 (76.3–102.8)	95.0 (79.0–107.0)	86.0 (76.0–96.0)	90.0 (76–102)
Hct, %	36.2 (34.3–37.8)	34.6 (32.9–36.3)	35.7 (33.9–37.1)	37.1 (34.7–38.7)	36.0 (34.0–38‐1)	36.3 (33.9–39.4)	35.4 (33.2–37.6)	36.4 (34.7–38.5)	37.3 (35.8–40.7)	36.2 (34.3–38.5)
RBC mass, mL	32.0 (28.7–37.2)	37.0 (32.0–42.0)	32.5 (24.5–41.5)	33.0 (30.2–39.2)	31.0 (25.8–36.2)	25.0 (19.8–31.0)	32.0 (27.3–37.0)	34.0 (28.0–39.0)	33.0 (27.3–37.0)	33.0 (27.0–38.0)
Hb, g/dL	11.3 (10.9–12.0)	11.4 (10.6–12.0)	11.3 (10.8–11.7)	11.9 (11.0–12.6)	11.0 (10.0–11.6)	11.7 (10.3–12.6)	11.1 (10.3–13.2)	11.7 (11.1–12‐7)	11.5 (10.9–12.5)	11.4 (10.8–12.2)
Leukocytes, 10^9^/L	11.1 (10.1–13.0)	9.3 (7.7–10.9)	10.1 (9.6–12‐8)	9.4 (7.2–11.6)	10.0 (8.2–11.4)	10.8 (9.3–12.5)	10.1 (8.9–11.9)	10.8 (8.4–12.8)	10.3 (9.0–12.2)	10.3 (8.7–12.0)
Platelets, 10^9^/L	220 (191–260)	202 (178–224)	221 (201–255)	195 (160–243)	201 (167–227)	216 (155–246)	203 (159–226)	240 (204–282)	225 (195–277)	214 (183–250)
Compliant for starting volume^a^	30 (100)	47 (100)	24 (80)	20 (100)	98 (100)	11 (78.6)	25 (100)	84 (100)	98 (95.1)	439 (96.)
Positive at microbial testing	0 (0)	1 (2.3)	3 (10.3)	0 (0)	8 (8.3)	1 (7.1)	0 (0)	0 (0)	5 (4.9)	18 (3.9)

Typed units	*N* 30	*N* 47	*N* 30	*N* 20	*N* 98	*N* 10	*N* 25	*N* 75	*N* 103	*N* 438
O positive	20 (66.7)	35 (74.5)	16 (53.3)	7 (35.0)	65 (67.7)	4 (40.0)	9 (36.0)	49 (65.3)	67 (65.0)	273 (62.3)
O negative	3 (10)	6 (12.8)	4 (13.3)	1 (5.0)	6 (6.3)	0 (0)	1 (4.0)	9 (12.0)	6 (5.8)	36 (8.2)
A positive	7 (23.3)	5 (10.6)	7 (23.3)	7 (35.0)	17 (17.3)	4 (40.0)	7 (28.0)	8 (10.7)	24 (23.3)	86 (19.6)
A negative	0 (0)	0 (0)	1 (3.3)	1 (5.0)	5 (5.1)	1 (4.0)	4 (16.0)	3 (4.0)	3 (2.9)	18 (4.1)
B positive	0 (0)	0 (0)	2 (6.6)	2 (10.0)	4 (4.1)	0 (0)	3 (12.0)	5 (6.7)	3 (2.9)	19 (4.3)
B negative	0 (0)	1 (2.1)	0 (0)	2 (10.0)	0 (0)	1 (10.0)	1 (4.0)	1 (1.3)	0 (0)	6 (1.3)

Abbreviations: Hb, hemoglobin; Htc, hematocrit; RBC, red blood cells; WBC, white blood cells.

^a^ To be eligible for fractionation, cord blood units should have a starting volume >67 mL with a hematocrit >32%. Data are expressed as median (25th to 75th percentile range) or *n* (%).

During the study, a total of 458 RBC units were transfused to 118 out of 142 enrolled patients: 240 to 60 neonates in the control arm and 218 to 58 patients in the intervention arm. Notably, 431 (94.1%) transfusions were administered before 32 weeks of age, and 402 (87.8%) before 30 weeks. In total, 351 transfusions were A‐RBCs and 107 CB‐RBCs. CB units were released at a median age of 7 days (IQR 5–11) and were irradiated at distribution. Regarding transfusions given before 30 weeks of age, which marked the end of the intervention period, patients in the experimental arm received 80 CB‐RBCs and 67 A‐RBC units. These latter were administered because no CB‐RBC units were available at the time of the transfusion request, despite the large number of units processed. As reported, the BORN study outcomes were significantly impacted by these protocol deviations.^13^ In fact, 24 (42.8%) out of 56 evaluable patients in the intervention arm received also A‐RBCs, explaining the similar incidence of severe retinopathy at intention‐to‐treat analysis.^13^

### 3.2 Main characteristics of CB units and quality parameters of CB‐RBC concentrates

Table 1 illustrates the characteristics of the 451 CB units at collection and their ABO/Rh typing distribution. Median volume at collection was 90 mL (IQR 76–102), Hct 36.2 (IQR 34.3–38.5), and Hb concentration 11.4 g/dL (IQR 10.8–12.2). A total of 437 (96.9%) units met protocol‐defined volume and Hct criteria (volume >67 mL and hematocrit [Htc] >32%), and 14 (3.1%) did not, with a different distribution among centers (*p* < 0.001 at χ^2^ test).

Leukodepletion was performed by filtering whole CB units before fractionation. The BioR Flex filter, marketed for RBC concentrates, was used for this purpose due to its smaller size compared to whole‐blood filters. Moreover, preliminary data showed that it resulted in a better RBC mass recovery than filtering RBC concentrates, as we had performed in previous studies.^11^, ^12^ Prior to study initiation, the off‐label use of the BioR Flex filter was validated at the coordinating center and the results are summarized in Table 2. A total of 40 CB units with a median volume of 78 mL (IQR 69–94) and a median Hct of 37.3% (IQR 34.7–39.2) were filtered in an average time of 3.8 min (IQR 3.6–4.3). Filtration resulted in a median volume loss of 27% (IQR 23–32), with a median post‐filtration volume of 58 mL (IQR 48–73) (Table 2). The recovery of the RBC mass was 72.3% (IQR 67.0–77.1). Leukodepletion was evaluated by flow cytometry count of residual white blood cells (WBCs) in 31 CB units. Results showed a 99.9% reduction (IQR 99.9–99.9) of the WBC content, and a median residual per unit of 0.014 × 10^6^ WBCs (IQR 0.005–0.036). In total, 30 out of 31 evaluated units (96.8%) in the validation protocol had a WBC residual <1 × 10^6^ (Table 2). Filtration also resulted in a substantial reduction in platelet content, removing 88.8% (IQR 84.1–90.7) of the initial platelets.

**TABLE 2:** Pre‐study validation of leukodepletion.

	CB units
	*N* 40
Pre‐filtration volume, mL	77.1 (70.4–94.3)
Pre‐filtration Hct, %	37.3 (34–7‐39.2)
Pre‐filtration RBC mass, mL	29.0 (24.8–34.3)
Pre‐filtration WBCs, 10^6^/unit	754 (606–998)
Pre‐filtration PLT, 10^9^/unit	17.4 (14.4–20.7)
Post‐filtration volume, mL	55.7 (47.6–72.9)
Volume loss, %	27.3 (22.9–32.0)
Post‐filtration Hct, %	36.0 (34.2–38.9)
Post‐filtration RBC mass, mL	21.0 (16.7–27.2)
RBC mass recovery, %	72.3 (67.0–77.1)
Post‐filtration WBCs, 10^6^/unit^a^	0.014 (0.005–0.036)
WBC removal, %^a^	99.9 (99.9–99.9)
Compliant for residual WBC^a^	30 (96.8)
Post‐filtration PLT, 10^9^/unit	1.8 (1.4–2‐9)
PLT removal, %	88.8 (84.1–90.7)

Abbreviations: CB, cord blood; Htc, hematocrit; RBC, red blood cells; WBC, white blood cells.

^a^ Data relative to 31 CB units. Data are expressed as median (25th to 75th percentile range) or *n* (%).

Table 3 illustrates the main parameters of RBC concentrates fractionated during the BORN study. All units were processed within 44 h from collection, with a median interval of 26.0 h (IQR 20.4–35.8). RBC concentrates had a median volume of 39 mL (IQR 30–50), a median Hct of 56.1 (IQR 52.3–59.4), and a median Hb content per unit of 6.8 g (IQR 5.2–8.5). The RBC mass recovery in RBC concentrates was 64.6% (IQR 58.4–72.0) of the initial RBC mass and was slightly related to the volume (*r =* 0.099, *p* = 0.039) and RBC mass (*r* = 0.111, *p* = 0.021) at collection. As expected, the volume of CB‐RBC concentrates was proportional to the volume at collection (*r* = 0.705, *p* = 0.001), to the initial Htc value (*r* = 0.348, *p* = 0.001), and RBC mass (*r* = 0.747, *p* < 0.001). Similarly, the final Hb content of CB‐RBC concentrates was proportional to the volume of the unit at collection (*r* = 0.705, *p* < 0.001), to the Htc value (*r* = 0.348, *p* < 0.001), RBC mass (*r* = 0.783, *p* < 0.001), and Hb content (*r* = 0.793, *p* < 0.001).

**TABLE 3:** Processing data and quality controls of CB‐RBC units produced during the BORN study.

	A	B	C	D	E	F	G	I	H	TOT
	(*n* 30)	(*n* 47)	(*n* 30)	(*n* 20)	(*n* 98)	(*n* 14)	(*n* 25)	(*n* 84)	(*n* 103)	451
Interval form collection, hours	30.5 (20.7–38.8)	28.3 (26.0–30.4)	25.8 (24.0–32.6)	22.6 (17.0–37.9)	31.8 (21.1–37.9)	21.8 (1.0–40.6)	23.0 (19.2–29.5)	24.6 (18.4–32.0)	24.1 (18.8–36.0)	26.0 (20.4–35.8)
CB‐RBC volume, mL	39.5 (30.7–48.5)	44.0 (34.0–50.0)	53.0 (44.5–61.0)	35.0 (29.0–40.2)	32.5 (28.0–44.0)	32 (29.3–39.0)	46.5 (30.2–57.0)	42.0 (30.2–50.0)	38.0 (30.5–45.5)	39.0 (30.0–50.0)
Hct, %	57.6 (54.2–60.7)	58.0 (56.0–59.5)	47.5 (44.3–52.7)	57.0 (52.4–58.5)	56.0 (52.0–59.0)	53.9 (37.9–61.8)	49.0 (41.4–52.6)	55.0 (52.6–56.6)	58.3 (55.4–61.9)	56.1 (52.3–59.4)
RBC mass, mL	22.0 (18.0–27.5)	25.0 (20.0–29.0)	27.0 (20.0–32.0)	20.5 (15.3–22.0)	18.0 (14.0–24.0)	16.0 (10.5–22.0)	18.5 (13.5–26.25)	22.0 (16.2–28.0)	22.0 (17.0–27.0)	21.6 (17.0–27.0)
RBC mass recovery, %	68.6 (63.4–75.7)	65.8 (62.2–69.6)	84.4 (69.3–96.2)	57.5 (50.0–64.5)	59.4 (53.7–69.4)	64.0 (53.0–71)	62.4 (48.0–83.3)	62.8 (58.4–69.9)	67.7 (60.8–73.1)	64.6 (58.4–72.0)
Hb/unit, g	6.9 (5.5–8.6)	8.2 (6.4–9.4)	8.3 (6.2–10.3)	6.4 (5.3–7.2)	5.6 (4.3–7.6)	5.5 (4.7–6.9)	6.0 (4.0–8.9)	7.3 (5.3–9.1)	6.9 (5.2–8.5)	6.8 (5.2–8.5)
Platelets, 10^9^/unit	2.3 (1.3–3.3)	3.9 (2.7–5.1)	2.3 (0.8–4.1)	1.6 (0.9–2.5)	1.7 (0.9–3.0)	2.6 (1.0–53.7)	1.2 (0.9–2.6)	2.4 (1.5–4.4)	1.0 (0.5–1.9)	1.9 (0.9–3.2)
Platelet removal, %	87.4 (83.9–91.5)	82.5 (77.9–85.5)	87.8 (82.9–94.7)	90.4 (86.6–94.5)	89.7 (84.3–93.1)	87.0 (81.8–91.4)	90.9 (86.1–95.0)	88.2 (84.7–92.6)	94.1 (91.5–96.6)	89.6 (84.8–93.6)
Compliant for Htc values^a^	29 (96.7)	47 (100)	10 (33.3)	18 (90.0)	85 (86.7)	9 (64.2)	9 (36.0)	78 (92.8)	94 (91.2)	379 (84.0)
Compliant for residual WBC	30/30 (100)	45/46 (97.8)	29/29 (100)	20/20 (100)	95/95 (100)	9/9 (100)	24/24 (100)	76/76 (100)	9/9 (100)	337/338 (99.7%)
End‐stage hemolysis	0.23 (0.01–0.47)	0.24 (0.17–0.35)	0.39 (0.31–0.63)	0.51 (0.41–0.65)	0.33 (0.28–0.62)	NR	NR	0.45 (0.40–0.51)	0.29 (0.22–0.41)	0.39 (0.25–0.51)
Compliant/tested for end storage hemolysis^a^	10/10 (100)	7/8 (87.5)	20/22 (90.9)	16/17 (94.1)	17/17 (100)	NA	NA	18/19 (94.7)	30/30 (100)	117/123 (94.3)

*Note*: Only CB units collected <44 h were eligible for fractionation. Data are expressed as median (25th to 75th percentile range) or *n* (%).

Abbreviations: CB‐RBCs, cord blood‐red blood cells; Hb, hemoglobin; Htc, hematocrit; NA, not assessed; NR, not reported; WBC, white blood cell.

^a^ Quality requirements for CB‐RBCs included Htc between 50% and 70%, residual WBC <10^6^ per unit, and hemolysis rate after 14 days of storage below 0.8% of the initial Hb in 90% of the units evaluated.

The platelet content in CB‐RBC concentrates was 1.9 × 10^9^/unit (IQR 0.9–3.7), confirming that near 90% of the initial platelet content was removed after filtration. The interval between collection and processing seemed to influence the platelet reduction rate, with a larger decrease after longer storage (*r* = 0.153, *p* = 0.001).

Regarding the RBC concentrate quality requirements, 379 of 451 (84.0%) CB‐RBC units had post‐processing Hct values between 50% and 70%, with significantly different compliance rates among centers (*p* < 0.001 at χ^2^ test). Centers with higher volume activity produced CB‐RBC units with significantly higher Hct values (*r* ^*2*^ = 0.05 at linear regression analysis between units processed and Hct values of produced CB‐RBCs, *p* < 0.001), suggesting that center performances could be enhanced by expanding practice. CB units that did not meet protocol‐defined criteria at collection (volume >67 mL and Htc >32%) more frequently produced CB‐RBC concentrates with low Htc (64.3% of CB‐RBCs with Htc <50% vs. 12.6% among compliant units, *p* < 0.001). In all centers, more than 90% of tested CB‐RBC units had residual WBCs <1 × 10^6^ with no difference among them (*p* = 0.606 at χ^2^ test). Similarly, the end‐storage hemolysis rate was below 0.8% of the initial hemoglobin content in 117 of 123 (94.3%) investigated units, without differences among centers (*p* = 0.251 at χ^2^ test). The end‐storage hemolysis rate was not related to unit characteristics, including CB volume, interval between collection and processing, CB‐RBC unit volume, Hb content, and residual platelets and WBCs.

## 4 DISCUSSION

The rationale of the BORN trial revolutionizes the approach to the transfusion therapy of extremely preterm neonates.^13^ The core concept is to ensure that physiologically elevated HbF levels persist in these patients, even after RBC transfusions. The outcome assessment in patients allocated to the experimental arm of the BORN study was severely affected by a high rate of protocol deviations, with more than 40% also receiving standard RBC transfusions.^13^ As a result, the advantage of CB‐RBC transfusions did not emerge across the entire patient population (intention‐to‐treat analysis) but only in the subgroup of patients who were properly transfused according to the arm allocation (per‐protocol analysis).^13^ Despite these limitations, the potential clinical benefit of this novel transfusion approach for severe ROP and BPD has attracted significant attention.^17^, ^18^, ^19^, ^20^, ^21^ Sharing data on the 451 CB units collected and processed during the BORN study period, along with highlighting the challenges encountered, could help inform the design of future clinical trials exploring this transfusion approach.

As stated above, an important issue encountered in the BORN study was the insufficient availability of CB‐RBC units for patients in the intervention arm, which was unexpected given that 5467 CB units were collected in the same period at participating CB banks. Among the 451 CB‐RBC units fractionated during the study, only 107 were transfused, whilst for 67 transfusion requests for patients in the intervention arm there were no available CB‐RBC units. The large number of collected units that were not processed into RBCs suggests that many of them did not meet the protocol‐defined criteria. The BORN study inventory included only units collected in the previous 44 h: this interval was set according to the threshold of 48 h generally accepted for transplant units, and estimating a total processing duration of 4 h. Nevertheless, it cannot be excluded that also CB collected earlier could be suitable for transfusion and extending this window could improve availability without compromising product quality.^22^ An additional eligibility criterion for the processing was the volume. It is acknowledged that the timing of cord clamping significantly influences CB volume and cell content in both vaginal and cesarean section delivery, with either in utero or ex utero placenta collection.^23^, ^24^, ^25^, ^26^, ^27^ For vigorous, healthy neonates, WHO, ACOG, and NICE strongly recommend delaying cord clamping for at least 1 min, especially in low‐resource settings with limited access to iron‐rich foods.^28^, ^29^, ^30^ This approach was followed at all centers participating in the BORN study, and the median reported CB unit volume of 90 mL (IQR 76–102) confirms that delaying cord clamping as recommended is still compatible with the collection of a CB unit suitable for processing into RBC concentrates. However, it is conceivable that the availability of CB units suitable for transfusion may be substantially increased by improving collection methods. Interestingly, a recent study on CB solidarity donation illustrated novel approaches combining empathetic, story‐based care with efficient, waste‐reducing workflows, which can effectively improve trust, patient engagement, and staff satisfaction, leading to significantly higher donation rates and quality units.^31^ The fractionation method used in the BORN study produces a 25–30% loss of the initial CB unit volume during filtration. Considering a transfusion dose of 20 mL/kg,^32^ it was estimated that fractionating CB units with a minimum volume of 67 mL and an Htc value of 32% would yield CB‐RBC concentrates suitable for clinical use. As expected, CB‐RBC volume and total Hb content depended on the volume and RBC mass content of the starting units. Nevertheless, centers processing more units produced RBC concentrates with higher Htc, denoting that expanding practice may improve processing performances.

An additional issue significantly affecting the CB‐RBC inventory during the BORN study was the units' short shelf life. Overall, in vitro storage studies indicate that the shelf‐life of CB‐RBCs may be limited to 21 days.^33^, ^34^, ^35^ CB‐RBC units could not be released until negative bacterial test results were available, requiring 5–7 days. In addition, all RBC units transfused during the BORN study were irradiated at distribution; since units older than 14 days cannot be irradiated, this further limited the distribution time window to 7–9 total days. Pre‐storage leucocyte depletion of blood components is recommended to reduce adverse transfusion reactions, limit transfusion‐transmitted infections, and improve RBC storage.^36^ Whole CB filtration resulted in highly satisfactory leukodepletion, with more than 99% of units having less than 1 × 10^6^ residual WBCs, the limit still recommended by the most recent EDQM guidelines for leukocyte‐depleted additive solution RBC concentrates.^37^ Moreover, residual WBC were below 10^5^ cells/unit in more than 90% of cases. It is generally acknowledged that blood components can be considered CMV‐safe if they contain <5 × 10^6^ leucocytes/unit.^38^ Accordingly, a total WBC count <1 × 10^6^/unit is considered CMV‐safe and suitable for neonatal transfusion.^39^ Nevertheless, unless CMV infection occurs during pregnancy, CB‐RBCs convey no risk for CMV transmission by the donor. In contrast to blood components from adult individuals, residual WBCs in CB‐RBCs exhibit a naïve and tolerant phenotype, typical of neonatal cells.^40^ All these considerations question the need to irradiate CB‐RBC units to prevent transfusion‐associated graft‐versus‐host disease (TA‐GvHD).^41^ Neonates in general, and preterm neonates in particular, are considered at risk for TA‐GvHD due to anecdotal cases of TA‐GvHD described following intrauterine transfusion and neonatal exchange blood transfusion.^42^ Accordingly, Italian guidelines recommend γ‐irradiated blood components for these patients.^32^ An analysis of the last 10 years' reports from the UK national hemovigilance scheme, Serious Hazards of Transfusion (2010–2019), evidenced no TA‐GvHD among 956 incidents of failure to receive irradiated components, suggesting a protective role for modern processing methods.^43^ Considering these aspects, more recent guidelines on TA‐GvHD prevention omit irradiation for neonates without a known or suspected defect of cellular immunity.^44^ An additional aspect to avoid irradiation of CB units is that scarce data are available on the damage to irradiated CB‐RBCs,^33^ whereas it is well known that irradiation impairs the quality of adult RBCs, increasing hemolysis and impairing oxygen delivery.^45^, ^46^

CB‐RBC concentrates are a novel blood product and no specific quality standards are available in the current blood product quality guidelines.^37^ Apart from the Hct values, all nine centers in the BORN study met compliance for residual WBCs. Seven of them also complied with end‐storage hemolysis rates, whereas two centers did not report the data. Overall, these findings suggest that the processing method used in the BORN study might be suitable for scaling CB transfusion for a broader clinical use. The new European regulation for substances of human origin favors the introduction of innovative blood components adopting the GAPP (facilitatinG the Authorization of Preparation Process) methodology by European National competent authorities. GAPP includes a standardized assessment across member states to evaluate the safety and efficacy of new products, and harmonized procedures to determine the level of clinical evidence (from in vitro validation to clinical follow‐up) required to authorize the new preparation process.^47^ Indeed, to promote CB‐RBC transfusion for preterm neonates from a restricted experimental setting to wider clinical use, it will be necessary to obtain competent authority authorization through the GAPP methodology. The GAPP methodology uses the EuroGTP II Interactive Assessment Tool to determine the novelty and risks associated with new components or preparations, as well as the eventual measures to assure their safety and efficacy.^48^ According to available processing and clinical data, the EuroGTP II tool estimated that the use of this novel component has a low risk, which can be further minimized by careful monitoring of processing data and transfusion effects.^47^

Finally, it should be mentioned that during the BORN study we used eligibility criteria set by the Italian regulatory framework for donating CB hematopoietic stem cells.^15^ The assessment includes the evaluation of paternal and maternal clinical history and is focused on a wide range of hereditary and genetic diseases potentially transmitted by the graft. Eligibility criteria for donating CB for transfusion could be appropriately revised, limiting the assessment to maternal history and focusing on transfusion‐transmissible diseases. Hopefully, this simplification could lead to a greater propensity among pregnant women to donate CB.

In conclusion, CB‐RBC transfusion for ELGANs is supported by a robust biological rationale. Preliminary clinical data suggest that HbF exerts a physiological protective effect in these patients, while evidence regarding product quality and safety remains highly encouraging.^13^ In the future, operational refinements such as more rapid methods to detect microbial contamination or CB‐RBC‐specific pathogen inactivation technologies will undoubtedly improve the utilization of the CB inventory. Presently, the scalability of the CB processing, as designed in the BORN study, paves the way to next steps for an initial implementation of this transfusion approach under the precautionary monitoring of the National competent authority. Data from the present study suggest that hospitals with large patient populations and high CB collection capacity are likely better suited for the initial phase of the implementation. Moreover, increasing awareness among donors and midwives through targeted educational initiatives is essential to enhance the supply of CB units. Additionally, collaborating with neonatologists and preterm neonate parents' associations could prove instrumental in promoting the informed altruistic CB donation.

## CONFLICT OF INTEREST STATEMENT

The authors declare no conflicts of interest.

## Data Availability

The data that support the findings of this study are available from the corresponding author upon reasonable request.
